# IL-32α suppresses colorectal cancer development via TNFR1-mediated death signaling

**DOI:** 10.18632/oncotarget.3197

**Published:** 2015-04-13

**Authors:** Hyung-Mun Yun, Kyung-Ran Park, Eun-Cheol Kim, Sang Bae Han, Do Young Yoon, Jin Tae Hong

**Affiliations:** ^1^ Department of Maxillofacial Tissue Regeneration, School of Dentistry and Research Center for Tooth & Periodontal Regeneration (MRC), Kyung Hee University, Seoul 130-701, Republic of Korea; ^2^ College of Pharmacy and Medical Research Center, Chungbuk National University, Heungduk-gu, Cheongju, Chungbuk 361-763, Republic of Korea; ^3^ Department of Bioscience and Biotechnology, Bio/Molecular Informatics Center, Konkuk University, Seoul 150-716, Republic of Korea

**Keywords:** IL-32α, colon cancer, TNFR1, RIP1

## Abstract

Inflammation is associated with cancer-prone microenvironment, leading to cancer. IL-32 is expressed in chronic inflammation-linked human cancers. To investigate IL-32α in inflammation-linked colorectal carcinogenesis, we generated a strain of mice, expressing IL-32 (IL-32α-Tg). In IL-32α-Tg mice, azoxymethane (AOM)-induced colon cancer incidence was decreased, whereas expression of TNFR1 and TNFR1-medicated apoptosis was increased. Also, IL-32α increased ROS production to induce prolonged JNK activation. In colon cancer patients, IL-32α and TNFR1 were increased. These findings indicate that IL-32α suppressed colon cancer development by promoting the death signaling of TNFR1.

## INTRODUCTION

IL-32 is a novel cytokine, isolated from activated human natural killer (NK) cells or T cells [1–2]. IL-32α is the shortest over other IL-32 isoforms including IL-32β and IL-32γ, which exhibites higher biological activity, inducing cytokines, such as such as Tumour Necrosis Factor-α (TNF-α) and macrophage inflammatory protein 2 (MIP-2) [3]. IL-32β/γ isoforms were sufficiently detected into cell culture supernatants, whereas IL-32α was only in intracellular fraction, implying that it has potential roles as non-secreted cytokine although it is considered less potent than other IL-32 isoforms [4–5].

Colorectal cancer (CRC) is a major health problem and frequent cause of cancer mortality in the world [6]. It is the third most commonly diagnosed malignancy and the fourth most common cause of death worldwide [7–8]. The risk of CRC has been involved in the extent and duration of inflammation [9–10], which is a complex process of molecular and cellular signals, that cells release at the site of the injury to cause a number of changes: increased blood flow, exudation of fluids containing proteins like immunoglobulins, and infiltration by monocytes, granulocytes and lymphocytes. Although individual genetic background is associated with the risk of developing colorectal cancer, the most widely held belief is that the increased cancer risk is chronic inflammation [11]. Supporting evidence is that extensive colitis is a risk factor for CRC [12]. The involvement of inflammatory cytokine IL-32 suggests a potential role in cancer development. In the previous study, we found that IL-32β inhibited melanoma, colon, and prostate cancer growth via the activation of cytotoxic T cells and NK cells [13]. In addition, the overexpression of IL-32β/γ may also enhance immune responses to the cancer via an increase of traffic into the cancer area or maturation of immune cells, especially cytotoxic T cells and NK cells [14]. Recently, another study showed that IL-32α overexpression inhibited human chronic myeloid leukemia via enhancement of NK cells killing ability of natural killer cells [15]. Thus, the present study for IL-32α in inflammation-linked cancers will present the understanding, the interesting, and the scientific meaning.

Tumor necrosis factor receptor (TNFR) plays a critical role in diverse cellular events, including cell proliferation, differentiation, apoptosis, and necrosis [16–17]. Since the discovery of its tumoricidal function that destroys tumor cells, a lot of studies for TNFR signaling have been undertaken [18–19]. The signaling of TNFR is mediated by TNFR1 and TNFR2, resulting in several opposing cellular functions [20–23]. TNFR1 has been shown to promote most often apoptosis, whereas TNFR2 has been shown to be critical in cell migration and proliferation [20–21, 24]. In studies of acute colitis, TNFR1 knockout mice showed development of aggressive dysplastic lesions with more severe inflammation, however, TNFR2 knockout mice did not affect dysplastic changes [24]. The association of TNFR1 with control of apoptosis via death domain-depend signaling, leading to recruiting TNFR1-associated death domoain protein (TRADD), receptor interesting protein 1 (RIP1), TNFR-associated factor 2 (TRAF2), and Fas-associated death domain protein (FADD), and impaired apoptosis is one of hallmarks of carcinogenesis [16, 18, 23, 25].

Many murine models of sporadic and inflammation-related colon carcinogenesis have been developed in the last decade, including chemically induced CRC [26, 27]. Although inflammation-linked carcinogenesis has been well appreciated, the underlying mechanisms that lead to cancer development in chronic intestinal inflammation remain to be elucidated. In the present study, we investigated whether IL-32 regulates inflammation-linked carcinogenesis using an azoxymethane (AOM)-induced CRC model.

## RESULTS

### Inhibition of colon tumor development in IL-32α transgenic mice

**Figure 1:**
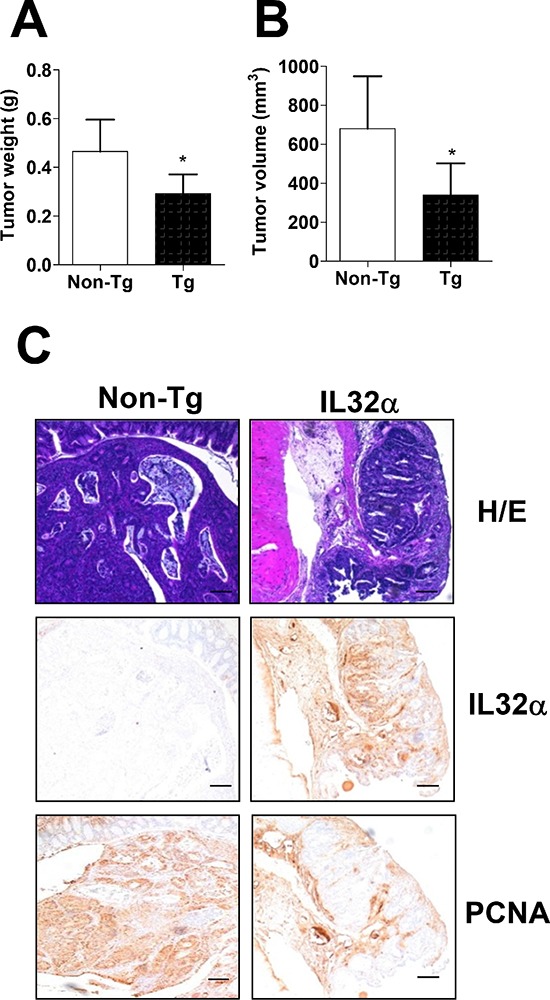
Inhibition of cancer development in IL-32α Tg mice **(A, B)** 7-week-old non-Tg and IL-32α-Tg mice were injected intraperitoneally with 10 mg/kg of AOM once a week for 6 weeks. Mice were killed at 32 weeks after AOM injections. Colons were harvested and Tumor weight (A) and volume (B) were monitored. **(C)** Colon tissues were processed and stained with H&E or analyzed by immunohistochemistry for detection of positive cells for IL-32α and PCNA. The images are representative of three separate experiments performed in triplicate. Scale bars indicate 100 μm. *Significant difference from non-Tg mice (*p < 0.05).

In order to investigate the functional role of IL-32α in colon carcinogenesis, we applied a murine colon carcinoma model based on the mutagenic agent, azoxymethan (AOM). 32 weeks after the start of the AOM injections, the mice were sacrificed and monitored. The tumor weight (Figure 1A, *p < 0.5) and volume (Figure 1B, *p < 0.5) in IL-32α Tg mice were significantly decreased compared with those in non-Tg mice. The immunohistochemical analysis of tissue sections was stained with H&E, and proliferating cell nuclear antigen (PCNA) also revealed greater inhibition of tumor growth in IL-32α Tg mice (Figure 1C). Furthermore, immunohistochemical analysis also showed that numbers of IL-32α immunoreactive cells were higher in the tissues of IL-32α Tg mice compared to that in non-Tg mice (Figure 1C).

### Upregulation of TNFR1 and induction of cell death in cancer tissue of IL-32α transgenic mice

**Figure 2:**
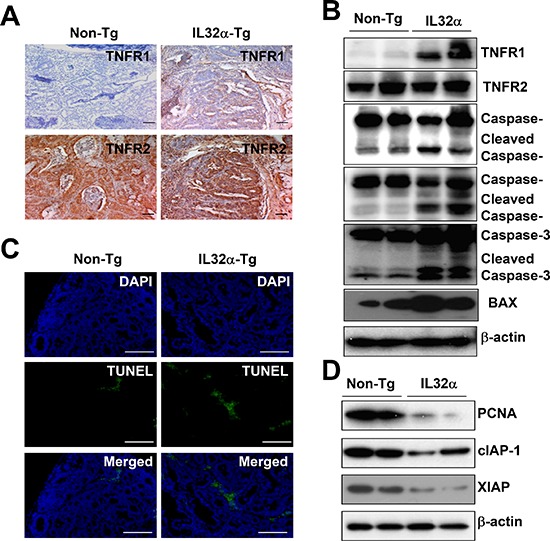
Expression of TNFR1 and cell death in colon cancer tissues of IL-32α Tg mice **(A)** TNFR1 and TNFR2 were observed by immunohistochemical analysis as described in Materials and Methods. **(B)** The expression of TNFR1, TNFR2, apoptotic proteins were detected by Western blotting using specific antibodies in tumor tissue extracts. β-actin protein was used as an loading control. **(C)** Apoptotic cells were examined by TUNEL staining. **(D)** Cancer extracts were analyzed by Western blotting as described in Materials and Methods section. Each images and band are representative of three independent mice.

Immunohistochemical staining of colon tumor tissues showed upregulation of TNFR1 expression in IL-32α Tg mice compared to the tumor tissues in non-Tg mice, while there was no difference of the expression of TNFR2 between non-Tg mice and IL-32α Tg mice (Figure 2A). Consistent with immunohistochemical staining, the upregulation of TNFRI was also confirmed by Western blot analysis (Figure 2B). TNFR1 primarily initiated cell death pathway, via caspase cleavage. As shown in Figure 2B and 2C, the data also showed that the expression of cleaved caspase-8, -9, -3, Bax, and TUNEL-positive cells was increased in the cancer tissues of IL-32α Tg mice than in those of non-Tg mice. On the contrary, PCNA, cIAP-1, and XIAP were decreased in tumor tissue lysates from IL-32α Tg mice compared to non-Tg mice (Figure 2D). These data suggested that IL-32α inhibits AOM-induced carcinogenesis by upregulating TNFR1 levels.

### IL-32α inhibits colon cancer cell growth and increases TNFR1-mediated cell death signaling

**Figure 3:**
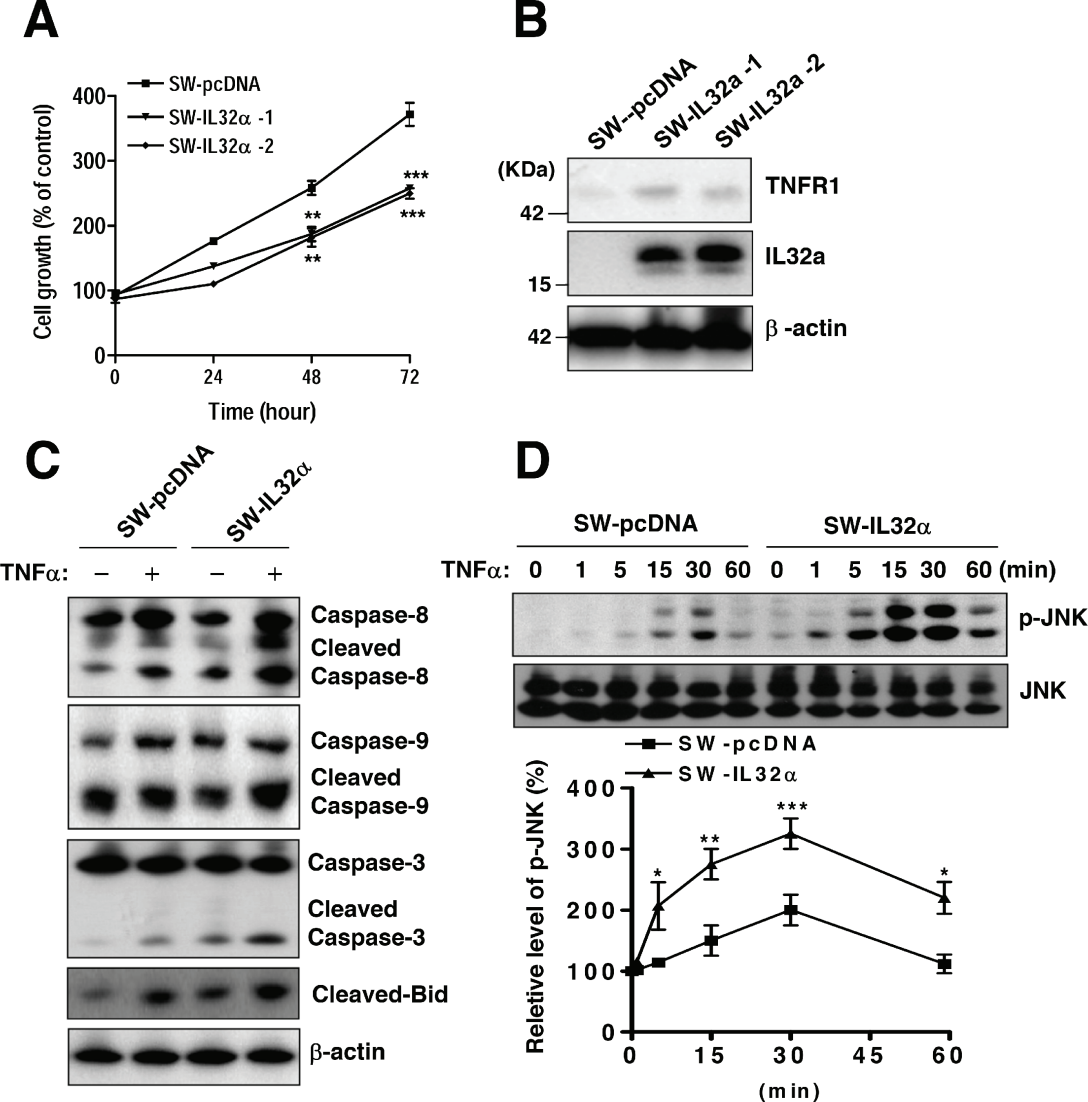
Effects of stable expression of IL-32α in SW620 cells on colon cancer cell growth and apoptotic signaling **(A)** SW620 cells were stably transfected with either the empty pcDNA3.1 vector (SW-pcDNA cells) or the IL-32α expression vector (SW-IL-32α cells), respectively. Cell growth rate was measured by MTT assay during 72 hr **(B)** Expression of IL-32α and TNFR1 is shown by Western blot analysis. β-actin protein was used as an loading control. **(C)** Cells were treated with 30 ng/ml TNFα for 24 hr. Cell extracts were analyzed by Western blotting using specific antibodies. **(D)** The cells were treated with TNFα (30 ng/ml) for the indicated times and assayed to detect phospho-JNK and JNK. The data are represented as relative percentages of the control. *Significant difference from SW-pcDNA cells (*p < 0.05, **p < 0.01, and ***p < 0.001). Representative results shown in Figure 3 were repeated in triplicate with similar results.

Next, it was validated whether IL-32α inhibits colon cancer cell growth. Consistent with IL-32α Tg mice, the stable expression of IL-32α in SW620 cells (SW-IL-32α cells) was significantly inhibited cell growth in a time dependent manner (Figure 3A). It was also found that SW-IL-32α cells have higher TNFR1expression level compared to SW-pcDNA cells (Figure 3B). To confirm the promoting effect of IL-32α on TNFR1-mediated cell death, it was investigated for the expression of apoptotic regulatory proteins in SW-IL-32α cells. As shown in Figure 3C, in the IL-32α-cells stimulated by TNF-α, cleaved caspases-8, cleaved caspases-9, cleaved caspases-3, and cleaved-Bid were more increased compared to TNF-α-stimulated SW-pcDNA cells. Next, it was evaluated for the underlying signal transduction pathway of IL-32α during TNF-α-induced cell growth inhibition. In colon cancer cells, prolonged JNK activation via TNFR1 is involved in growth arrest and cell death. Thus, it was investigated whether the pathways are regulated by IL-32α in TNF-α-mediated cell death. SW-IL-32α cells were treated with TNF-α for 0, 1, 5, 15, 30, and 60 min and the activation of JNK (phospho-JNK) was measured. TNF-α-induced phosphorylation of JNK was significantly increased at 15 min, reached a maximal response at 30 min and returned to a basal level after 60 min incubation in SW-pcDNA cells. While, in SW-IL-32α cells with TNF-α, the activation of JNK (phospho-JNK) was significantly higher, and started to increase significantly at 1 min, reached a maximal response at 30 min, and sustained until 60 min (Figure 3D). In reverse proportion to JNK activation, it was found that NF-κB signaling was significantly inhibited in SW-IL-32α cells (Supplementary Figure 1). These data suggest that IL-32α enhances TNFR1-mediated cell death signaling.

### IL-32α elevates ROS production to induce prolonged JNK activation

**Figure 4:**
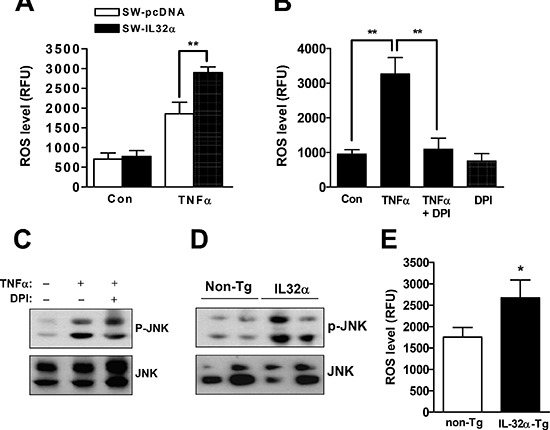
Effects of IL-32α on ROS release and JNK activation in cancer tissues and colon cancer cells **(A)** Cells were treated with 30 ng/ml TNFα for 4 hr in SW-pcDNA cells and SW-IL-32α cells. *Significant difference from SW-pcDNA cells (**p < 0.01). **(B)** SW-IL-32α cells were treated with TNFα for 4 hr in the absence or presence of the NOX inhibitor, DPI (20 μM) for 30 min. ROS levels were determined using ROS detection kit as described in Materials and Methods section. *Significant difference from control or TNFα-treated cell (**p < 0.01). **(C)** After SW-IL-32α cells were treated with TNFα for 60 min in the absence or presence of DPI (20 μM) for 30 min, cell extracts were analyzed by Western blotting using anti-phospho-JNK and anti-JNK antibodies. **(D, E)** Cancer extracts were analyzed by Western blotting (D) and ROS detection kit (E) as described in Materials and Methods section. *Significant difference from non-Tg mice (**p < 0.01). Representative results shown in Figure 4 were repeated in triplicate with similar results.

Sustained JNK activation appears to correlate with ROS generation and cell death. Thus, it was further investigated whether IL-32α elevated ROS generation in colon cancer. As shown in Figure 4, SW-IL-32α cells significantly increased ROS level (Figure 4A), which was prevented by an inhibitor of nicotinamide adenine dinucleotide phosphate-oxidase (NOX), DPI (Figure 4B). The pretreatment of DPI also inhibited prolonged JNK activation (phospho-JNK) (Figure 4C). It was validated that the knockdown of TNFR1 significantly attenuated ROS generation in SW-IL-32α cells (Supplementary Figure 2). In parallel with colon cancer cell growth, the phosphorylation of JNK (Figure 4D) and the level of ROS (Figure 4E) were also increased in IL-32α Tg mice compared to non-Tg mice. These data suggest that ROS-JNK signaling of TNFR1 is critical mediator of IL-32α-mediated anti-cancer effect.

### Pattern of IL-32α and TNFR1 expression in human colon cancer

**Figure 5:**
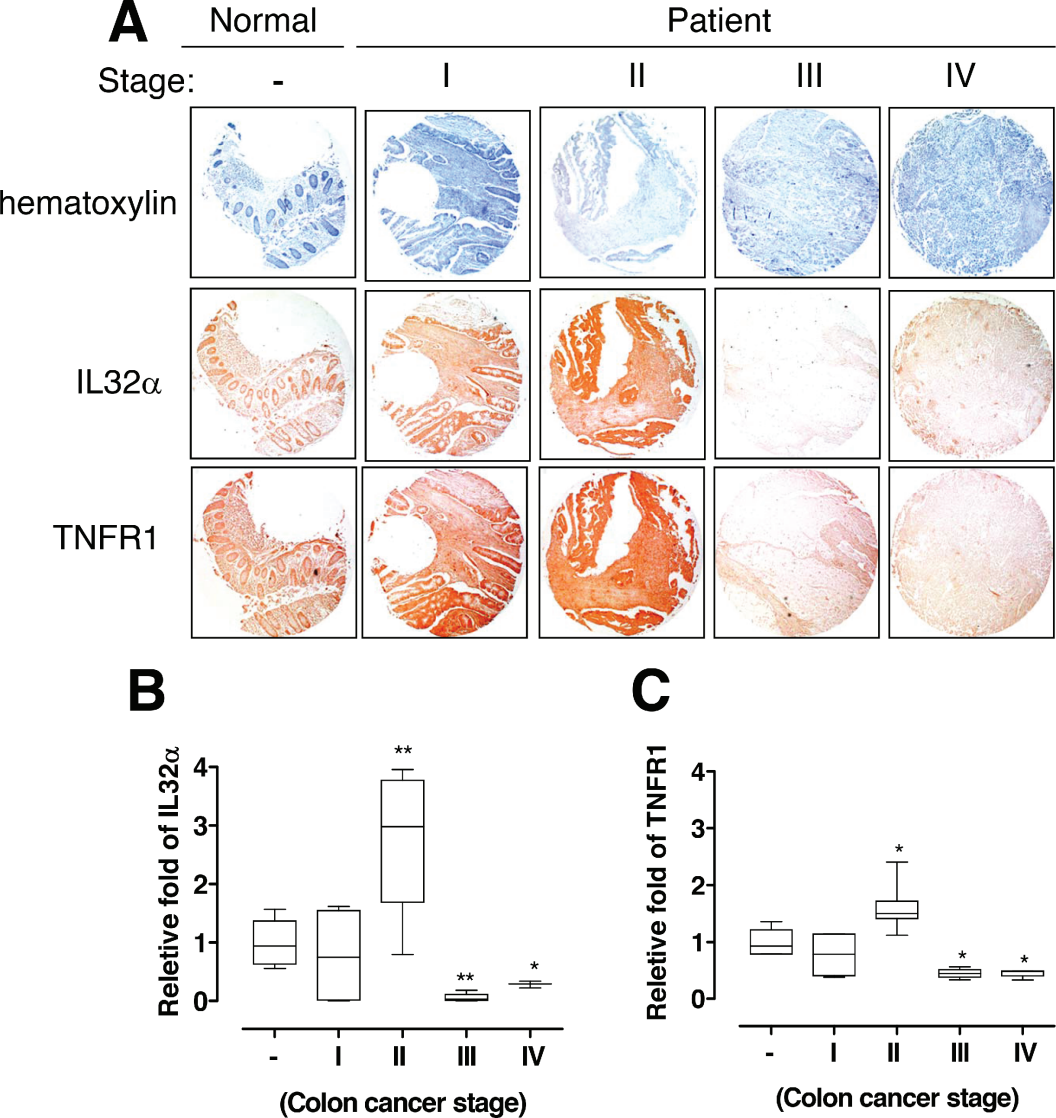
Relationship between IL-32α and TNFR1 in human colon cancer patients **(A)** Human normal colon or cancer sections (Stage I–IV) were processed and stained with Hematoxylin or analyzed by immunohistochemistry for detection of positive cells for IL-32α and TNFR1. **(B, C)** The values are releative fold from human normal colon sections against IL-32α and TNFR1 (C). *Significant difference from normal sections cells (*p < 0.05 and **p < 0.01).

To further determine the pathological relevance between IL-32α and TNFR1 expression in colon tumor patients, we examined whether the expression of IL-32α was related with TNFR1 expression using application of human colon tumor tissue microarray. In immunohistochemical staining, there was a significant correlation between the IL-32α and TNFR1 (Figure 5A). Interestingly, IL-32α and TNFR1 expression were increased until stage II, whereas the expression was eventually decreased in stage III and IV in human colon tumor tissue when compared to normal colon tissues (Figure 5B and 5C). Thus, these data support the reliability that IL-32α and TNFR1 may dynamically play a role in the development of human colon cancer.

### IL-32α increases TNFR1-adaptor complex in human colon cancer

**Figure 6:**
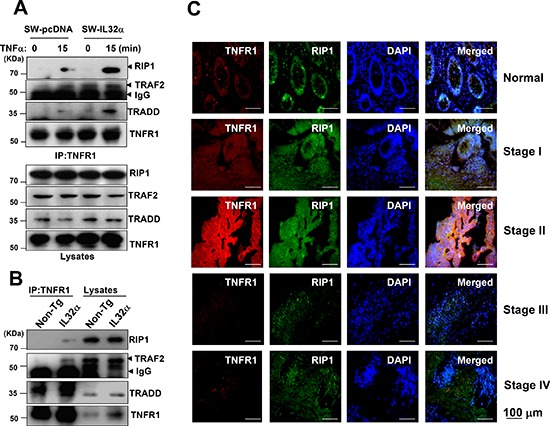
Effect of IL-32α on TNFR1-adaptor complex in colon cancer development **(A, B)** SW-pcDNA cells and SW-IL-32α cells were treated with 30 ng/ml TNFα for 15 min. The colon cancer cells (A) and colon tumor tissues (B) from non-Tg and IL-32α Tg mice were lysated, and then immunoprecipitated with anti-TNFR1 antibody. The immunocomplexes were analyzed by immunoblotting with anti-RIP1, anti-TRAF2, anti-TRADD, anti-TNFR1 antibodies. The total protein expression in cell lysates was identified with the specific antibodies. **(C)** After human patient tissues were permeabilized, TNFR1 (*red*) was immunostained with mouse anti-TNFR1 followed by Alex555-conjugated secondary antibodies and RIP1 (*green*) was immunostained with rabbit anti-RIP1 antibody, followed by Alex488-conjugated secondary antibodies. And then sections were stained with DAPI (*blue*). The *right panels* show the merged images of the *first, second*, and *third panels*.

The next question was if IL-32α was required for a change in the TNFR1-adaptor complex that brings about the binding of the TRADD, TRAF2, and RIP1 adaptor proteins to the death domain of the TNFR1. In SW-IL-32α cells, TNFR1 led to increased association of a signaling complex including TRADD, RIP1, and TRAF2 compared to SW-pcDNA cells (Figure 6A). In addition, the complex was increased in colon tumor tissues of IL-32α Tg mice compared to those of non-Tg mice (Figure 6B). As shown in Figure 6C, the association of TNFR1 with RIP1 was increased until stage II, whereas the association was decreased in stage III and IV in parallel with the expression pattern of IL-32α and TNFR1 (Figure 5) when compared to normal tissues. Overall our data indicate that IL-32α plays a role as a regulator of signaling complex of TNFR1 in the development of human colon tumor.

## DISCUSSION

In present study, we demonstrated that the expression of IL-32α concomitantly increased the signaling complex of TNFR1 with TRADD, TRAF2, and RIP1 in colon cancer cell, AOM-induced CRC mice, and colon cancer patient tissues. Subsequently, the expression of IL-32α inhibited colon cancer cell growth and suppressed colorectal cancer development via ROS, JNK, and caspase signaling. TNFR1 has been shown to promote cell death and reduce inflammatory damages [20, 28–29]. The signaling of TNFR1 is initiated by recruitment of cytosolic proteins through protein-protein interaction domains in cytoplasmic death domain which TNFR2 does not contain. TNFR1 recruits a key adaptor protein, TRADD, which serves as a supporting structure for recruitment of TRAF2, RIP-1, FADD, and caspase 8 essential for TNFR1-induced cell death [16, 25, 30]. These data suggest that IL-32α is closely related to lead to TNFR1-mediated cancer cell death in colorectal cancer development. Previous studies discovered the critical roles of ROS in TNFR1-mediated cell death signaling, in particular on sustained JNK activation [30–32]. It is now recognized that there are two phases of JNK activation mediated by two different activation pathways. The earlier and transient activation of JNK is mediated by TRAF2 [33], whereas the delayed and persistent activation of JNK is mediated by ROS [34–35]. We demonstrated that IL-32α induced JNK activation and increased ROS production through TNFR1. ROS was also required for JNK activation, which further contributed to ROS production (Supplementary Figure 3). Evidences supporting such notion include that ROS promotes TNFR1-induced sustained JNK activation by inhibiting MAP kinase phosphatases [36] or activating activation of apoptosis signal-regulating kinase 1 (ASK1) that is an upstream protein of JNK and p38, leading to apoptosis (p38 was also activated by IL-32α, Supplementary Figure 4) [37–38]. Although it is not known how IL-32α promotes ROS production, it seems that there is a positive feedback loop between JNK activation and ROS production, and both work together contributing to TNFR1-induced cell death.

The expression of TNFR1 reported prognostic relevance in head and neck cancer (HNC) patients and from patients with other primary carcinomas and sarcomas [39]. Especially, the expression pattern and prognostic impact of TNFR1 in colorectal cancers were reported [40]. Patients with a high TNFR1 expression had a significantly better survival rate than those with a low TNFR1 expression [40]. In the present study, IL-32α Tg mice showed higher TNFR1 expression in AOM-induced CRC tissues. In human colon tumor patient, the expression pattern of IL-32α was also correlated with the expression pattern of TNFR1. Interestingly, the expression level was significantly increased until stage II but drastically decreased in stage III/IV. To our knowledge, the expression pattern suggest that the progression into aggressive cancer stages is due to the loss of anti-cancer effect via suppression of the expression of TNFR1 and IL-32α, and the cell death signaling of TNFR1. Chang and colleague reported that the loss of TNFR1 signaling using TNFR1 knock-out mice showed weight loss, severe inflammation, significantly increased vulnerability to carcinogenesis [24]. Similarly, we also demonstrated that the overexpression of IL-32α upregulated TNFR1 and inhibited CRC development in IL-32α Tg mice and cell lines. Therefore, it suggests that the regulation of IL-32α expression is an important factor for CRC development.

In conclusion, we demonstrate the inhibitory effect of IL-32α on colon tumor provide novel evidence that IL-32α has a suppressive effect on colon tumor growth through the positive regulation of TNFR1-mediated death signaling during CRC development.

## MATERIALS AND METHODS

### Animals

Non-Tg mice (*n* = 10) and IL-32α Tg mice (*n* =10) were used. At 7 weeks of age, all animals except those intended for saline were injected intraperitoneally with 10 mg/kg of AOM once a week for 6 weeks and sacrificed by CO_2_ euthanasia at 32 weeks after the start of the AOM injections.

### Plasmids

To generate transgenic mice that express hIL-32α, concentrated hIL-32α cDNA was prepared. The human IL-32α gene (hIL-32α cDNA) was subcloned into the *Eco*RI sites of the pCAGGs expression vector from pcDNA3.1 vector. The IL-32α gene-containing genomic fragment was isolated after restriction enzyme digestion with SalI/HindIII from the vector, and then separated by a low melting-point agarose from the pCAGGs vector using transverse alternating field-gel electrophoresis.

### IL-32α-Tg mice

The hIL-32α cDNA fragment was purified and microinjected into the embryos of BDF1 mice. The experimental treatments were carried out according to the guidelines for animal experiments of the Faculty of Disease Animal Model Research Center, Korea Research Institute of Bioscience and Biotechnology (Daejeon, Korea) as well as the guidelines for the welfare and use of animals in cancer research. Diethyl ether was used in animal experiments as a euthanasia agent. After appropriate quantity of ether was poured onto cotton wool and allowed to evaporate and fill the chamber, the animals were placed on the ether soaked cotton wool under a mesh so that the animals do not have direct contact with the liquid chemical (ether). After euthanasia, the animal carcasses and the soaked cotton wool were removed and placed inside the chemical fume hood to allow dissipation of the chemical. The extraction system of the fume hood remained switched on for a further period of 30 minutes after the animal carcasses were handled following standard clinical waste procedures.

### Genomic DNA and PCR

IL-32α insertion was confirmed by the amplification of genomic DNA isolated from the transgenic mice tails using Super Taq PLUS Pre-mix (RexGene BioTech, Korea) and the following specific primer set: sense, 5-GAA GGT CCT CTC TGA TGA CA-3; and antisense, 5-GAA GAG GGA CAG CTA TGA CTG-3 (nt 2245-2225). GAPDH was used as an internal control. Genomic DNA samples were extracted from transgenic mice tails using G-spin Total DNA Extraction Kit (iNtRON Biotechnology, Inc., South Korea) and PCR analysis was performed for detection of insertion of IL-32α in the genome. The following conditions were used for the TaKaRa PCR Thermal Cycler: 94°C for 10 min, followed by 35 cycles of 94°C for 30 sec, 63°C for 30 sec, and 72°C for 60 sec; with a final step of 72°C for 5 min.

### Ethics statement

All animal experiments were approved and carried out according to the Guide for the Care and Use of Animals [Chungbuk National University Animal Care Committee, Korea (CBNUA-045-0902-01)].

### Histological analysis

### Tissue samples treatment

Tissues were fixed in 10% buffered formalin, embedded in paraffin blocks, and processed for histological evaluation by routine procedures with H&E staining.

### Immunohistochemistry

Five-micrometer-thick tissue sections and colon cancer patient tissue array with normal colon tissues as control (US Biomax Inc., Rockville, MD) were mounted on poly-glycine-coated slides. The sections were deparaffinized by immersing into xylene solution, rehydrated, subjected to heat-mediated antigen retrieval treatment, washed with distilled water and proceed with immunohistochemical procedure. Endogenous peroxidase activity was quenched by incubation with 1% hydrogen peroxide solution in methanol for 30 min and washed with 1X PBS (Sigma, St. Louis, MO) for 5 min. Next, the sections were blocked with 3% normal horse/goat serum (Vector Laboratories, Burlingame, CA) diluted in 1X PBS for 30 min, incubated overnight with specific antibodies against IL-32α (1:100, BioLegend, San Diego, CA), PCNA (1:500; Cell signaling Technology, Beverly, MA, TNFR1 (1:400; Santa Cruz Biotechnology, Santa Cruz, CA), and TNFR2 (1:400; Santa Cruz Biotechnology) at 4°C, and washed 3 times with PBS 1X. The immunological detection was started with incubation in biotinylated goat anti-mouse/rabbit IgG antibody (1:1000 dilution, Vector Laboratories, Burlingame, CA) for 1 hr at room temperature, 3 washings with PBS 1X, continued with avidin-conjugated peroxidase complex (ABC kit, 1:200 dilution, Vector Laboratories, Burlingame, CA) for 30 min at room temperature 3 washings with PBS 1X. Chromogen development was performed with 0.02% 3, 3′-diaminobenzidine tetrahydrochloride (DAB, Vector Laboratories, Burlingame, CA) and slides counterstained with H&E. Finally, sections were dehydrated with ethanol, cleared with xylene, and mounted with Permount (Fisher Scientific, Rockford, IL), and evaluated on a light microscopy (Olympus, Tokyo, Japan).

### Immunofluorescence

Sections were incubated overnight with specific antibodies against TNFR1 (1:500; Cell signaling Technology) and RIP1 (1:500; Cell signaling Technology) at 4°C. After then, the sections were incubated with an anti-rabbit secondary antibody labeled with Alexa-Fluor 488 (1:400 dilution, Invitrogen, Carlsbad, CA) or anti-mouse secondary antibody labeled with Alexa-Fluor 568 (1:400 dilution, Invitrogen) for 2 h at room temperature. Final images were acquired using a confocal laser scanning microscope (TCS SP2, Leica Microsystems AG, Werzlar, Germany).

### Cell culture and transfection

Human SW620 colon cancer cell lines were obtained from the American Type Culture Collection (Manassas, VA). Colon cancer cells were incubated with medium RPMI 1640 containing L-glutamine 1X supplemented with 10% fetal bovine serum (FBS), 1% 10000 U/ml penicillin and 10000 μg/ml streptomycin, at 37°C in 5% CO2 humidified air. All reagents were purchased from Invitrogen (Carlsbad, CA, USA). To establish constitutive expression systems of IL-32α, cancer cells were transfected with the pcDNA3.1 or pcDNA3.1-IL-32α vector using the Lipofectamine™ 2000 (Invitrogen). The transfected cells were selected with an antibiotic for neomycin resistance gene, G418 (800 μg ml, Sigma). G418-resistant cells were screened for 3 weeks, and single cell-expanded clones were obtained by serial dilutions. We confirmed the stable expression of IL32α in SW620 cells using the Western blot. The cells were transiently transfected with TNFR1 siRNA (Santa Cruz Biotechnology) or Control siRNA (Santa Cruz Biotechnology) per well using a mixture of siRNA and WelFect-EX Plus reagent in OPTI-MEM, according to the manufacturer's specification (WelGENE, Seoul, Korea).

### TUNEL assay

DNA fragmentation was examined by terminal deoxynucleotidyl transferase-mediated FITC–dUDP nick-end labeling (TUNEL). TUNEL assays were performed using the *in situ* Cell Death Detection Kit (Roche Diagnostics GmbH, Mannheim, Germany) according to the manufacturer's instructions. Briefly, 25-μm cryosections were fixed with 4% paraformaldehyde, treated with 0.1% NaBH4 and 0.1% Triton X-100, and incubated for at least 1 h with a reaction mix containing deoxynucleotidyl transferase and FITC–dUDP (Roche, Reinach, Switzerland). For 4′,6′-diamidino-2-phenylindole dihydrochloride (DAPI, Sigma) staining, slides were incubated in the dark at room temperature for 15 min with mounting medium for fluorescence containing DAPI. The tissues were then observed through a fluorescence microscope (Leica Microsystems AG, Wetzlar, Germany) and the nuclei were visualized by the DAPI staining.

### Western blot analysis

Tissues and cells were homogenized and lysed with lysis buffer (1X PBS, pH 7.4, 10 mM dithiothreitol, 1 mM EDTA, 1% Triton X-100, and protease inhibitor cocktail) containing 2 mM Na_3_VO_4_, 10 mM Na_4_P_2_O_7_, and 10 mM NaF) for 1 hr on ice. The lysate was centrifuged at 14,000 rpm for 15 min at 4°C. Total protein concentration was determined by the Bradford method (Bio-Rad Laboratories, Berkeley, CA). An equal amount of total protein (20 μg) was resolved on an sodium dodecyl sulfate (SDS, Sigma) 10 or 12% polyacrylamide gel in 1X SDS-PAGE Running buffer (25 mM Tris, 192 mM glycine, 0.1% SDS) and then transferred to a nitrocellulose membrane (Hybond ECL; Amersham Pharmacia Biotech, Piscataway, NJ). Blots were blocked for 1 hr at room temperature with 5% (w/v) non-fat dried milk in Tris-buffered saline Tween-20 (TBST: 10 mM Tris (pH 8.0) and a 150 mM NaCl solution containing 0.05% Tween-20). After washing, membranes were incubated at 4°C for overnight with specific primary antibodies against TNFR1, TNFR2, Caspase-8, Caspase-9, Caspase-3, BAX, PCNA, cIAP-1, XIAP, IL-32α, Bid, phospho-JNK, JNK, RIP1, TRAF2, TRADD, phospho-p38, p38, and β-actin. The blots were then incubated with HRP-conjugated anti-mouse or anti-rabbit antibodies (1:2000, Santa Cruz Biotechnology). Immunoreactive proteins were detected with the enhanced chemiluminescence (ECL, Amersham Pharmacia Biotech) western blotting detection system.

### Reactive oxygen species (ROS) generation

Intracellular accumulation of Reactive oxygen species (ROS) was monitored using OxiSelect™ Hydrogen Peroxide/Peroxidase Assay Kit (Cell Biolabs, Inc., San Diego, CA). To investigate ROS generation, the tissues and cells were homogenized in 1X Assay Buffer. The lysates were assayed according to manufacturer's procedure.

### Tissue microarray

Tissue microarray incorporating a series of colon cancers with matched normal tissue (US Biomax Inc., Rockville, MD) was used with immunohistochemistry and Immunofluorescence.

### MTT assay

Cell viability was measured by performing an MTT (Sigma) assay to the detect dehydrogenase activity retained in living cells. An aliquot (50 μl) of MTT solution (5 mg/ml) in phosphate-buffered saline was directly added to the cultures, and the cultures were then incubated for 4 h to allow MTT to metabolize to formazan. Absorbance was measured with an automated spectrophotometric plate reader at a wavelength of 570 nm. Cell viability was expressed as relative percentages in comparison with controls.

### JNK activation

The cells were treated with TNFα (10 ng/ml) and lysed in lysis buffer (1X PBS, pH 7.4, 10 mM dithiothreitol, 1 mM EDTA, 1% Triton X-100, and protease inhibitor cocktail) containing 2 mM Na_3_VO_4_, 10 mM Na_4_P_2_O_7_, and 10 mM NaF. Equal amounts of lysate were prepared and immunoblotted with anti-JNK (1:1000; Cell Signaling Technology, Beverly, MA) and anti-phospho-JNK (1:1000; Cell Signaling Technology) antibodies.

### Co-immunoprecipitation

Cells and tissues were gently lysed with lysis buffer for 1 hr on ice and then centrifuged at 14,000 rpm and 4°C for 15 min, and the supernatant was collected. The soluble lysates were incubated with anti-TNFR1 antibody (Cell Signaling Technology) at 4°C and then with Protein A/G bead (Santa Cruz Biotechnology) and washed times. Immune complexes were eluted by boiling for 10 min at 95°C in SDS sample buffer followed by Western blot with anti-RIP1 (1:2000; Cell Signaling Technology), TRAF2(1:1000; Cell Signaling Technology), TRADD (1:1000; Cell Signaling Technology), or TNFR1 (1:2000; Cell Signaling Technology) antibodies.

### Statistical analysis

The data were analyzed using the GraphPad Prism version 4 program (GraphPad Software, Inc., San Diego, CA). Data are presented as mean ± SD. Statistical significance was performed on the data using one-way analysis of variance (ANOVA) or unpaired Student's *t*-test. A value of *p* < 0.05 was considered to be statistically significant.

## SUPPLEMENTARY FIGURES


