# Supplementary material for: IL-32α suppresses colorectal cancer development via TNFR1-mediated death signaling
Source: Oncotarget. 2015 Apr 13;6(11):9061–72. doi: 10.18632/oncotarget.3197 (PMC4496202; doi:10.18632/oncotarget.3197)
Supplement: Supplementary file 1 [file oncotarget-06-9061-s001.pdf]

SUPPLEMENTARY FIGURES

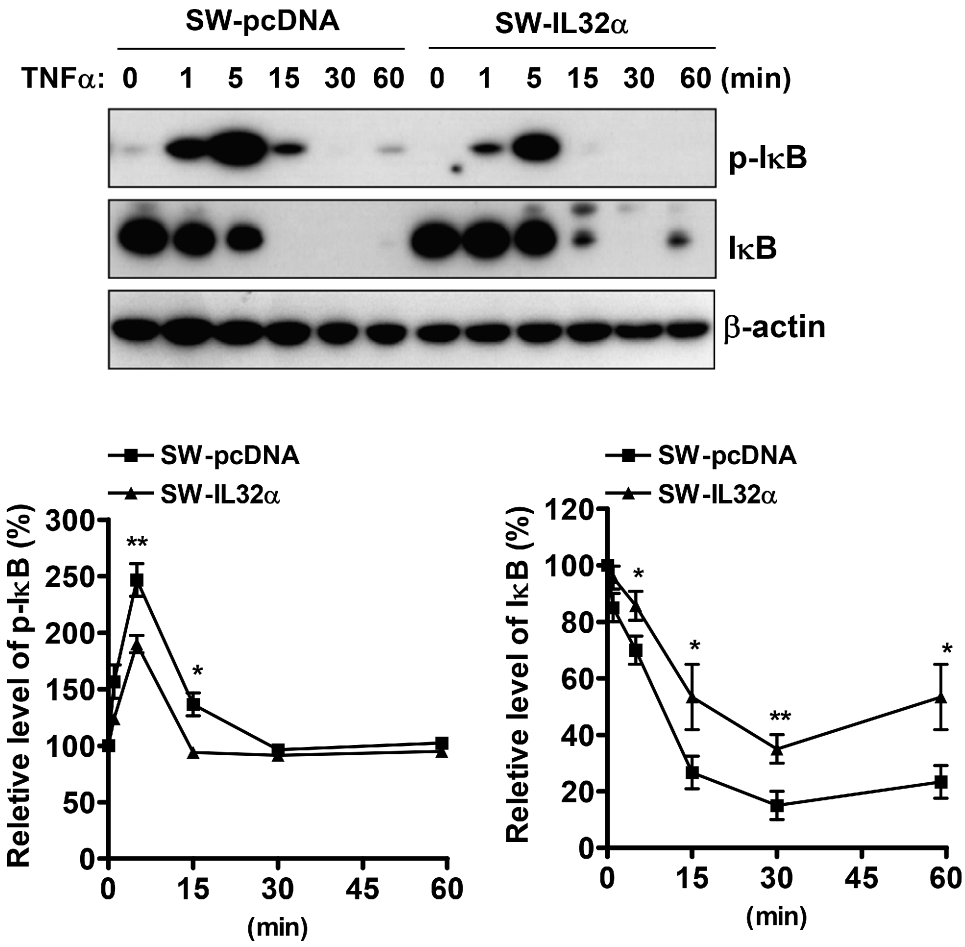

Supplementary Figure 1: Effects of IL-32α on NF-κB signaling in colon cancer cells. SW-pcDNA cells and SW-IL-32α cells were treated with TNFα (30 ng/ml) for the indicated times and assayed to detect phospho-IκB and IκB. The data are represented as relative percentages of the control. *Significant difference from SW-pcDNA cells (*$p < 0.05$ and **$p < 0.01$). Representative results shown in Supplementary Figure 1 were repeated in triplicate with similar results.

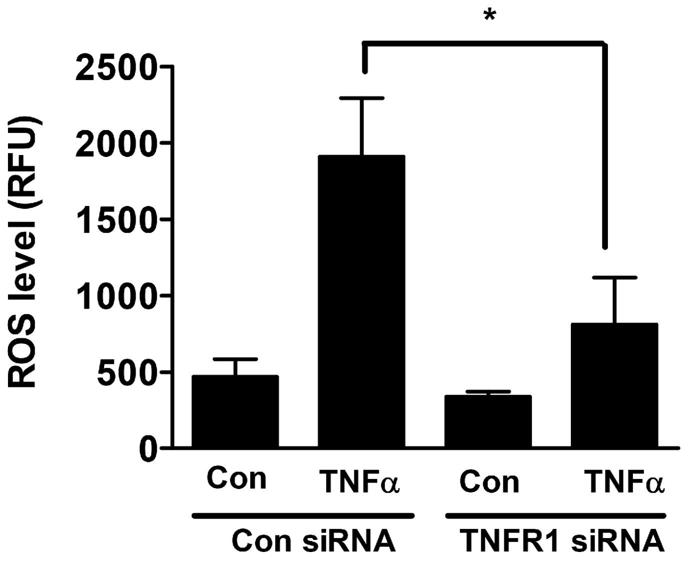

Supplementary Figure 2: Effects of knockdown of TNFR1 on ROS release in SW-IL-32α cells. (A) Cells were transfected with TNFR1 siRNA for 24 hr in the SW-IL-32α cells. After then, TNFα (30 ng/ml) were treated and ROS levels were determined as described in Materials and Methods section. *Significant difference from SW-pcDNA cells (**$p < 0.01$). Representative results shown in Supplementary Figure 2 were repeated in triplicate with similar results.

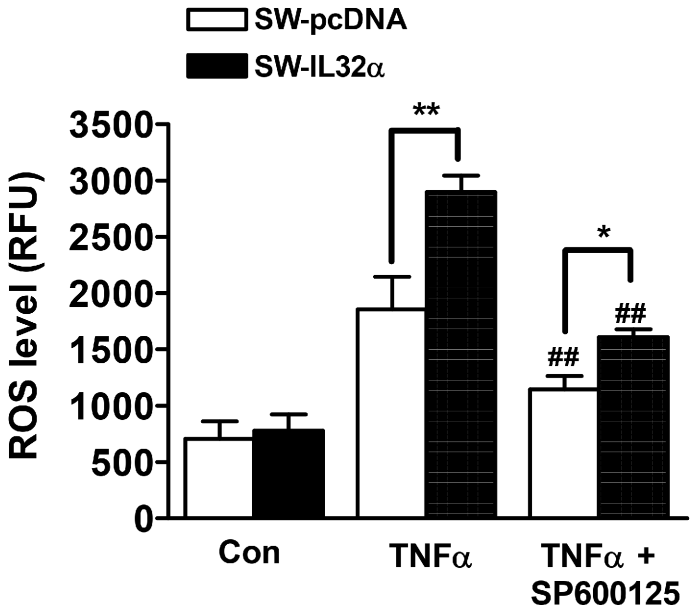

Supplementary Figure 3: Effects of JNK inhibitor on ROS release in colon cancer cells. SW-pcDNA cells and SW-IL-32α cells were treated with TNFα for 4 hr in the absence or presence of the JNK inhibitor, SB600125 (10 μM) for 30 min. ROS levels were determined using ROS detection kit as described in Materials and Methods section. *Significant difference SW-pcDNA cells (*$p < 0.05$ and **$p < 0.01$). #Significant difference from TNFα-treated cell (##$p < 0.01$). Representative results shown in Supplementary figure 3 were repeated in triplicate with similar results.

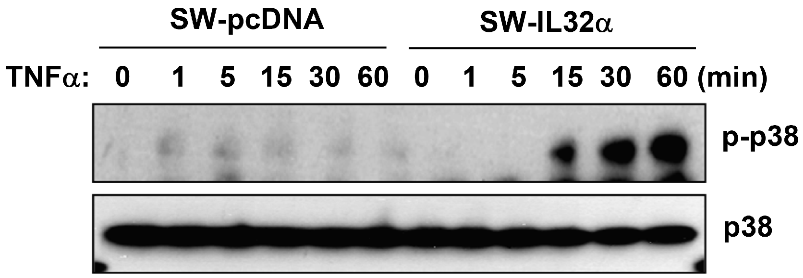

Supplementary Figure 4: Effects of stable expression of IL-32α in SW460 cells on phosphorylation of p38. SW-pcDNA cells and SW-IL-32α cells were treated with 30 ng/ml TNFα for the indicated times and then the activation of p38 was assayed using primary anti-phospho-p38 and anti-p38 antibodies. Representative results shown in Supplementary Figure 3 were repeated in triplicate with similar results.
